# CAGER: classification analysis of gene expression regulation using multiple information sources

**DOI:** 10.1186/1471-2105-6-114

**Published:** 2005-05-12

**Authors:** Jianhua Ruan, Weixiong Zhang

**Affiliations:** 1 Department of Computer Science and Engineering, Washington University, St. Louis, MO 63130, USA; 2 Department of Genetics, Washington University School of Medicine, St. Louis, MO 63110, USA

## Abstract

**Background:**

Many classification approaches have been applied to analyzing transcriptional regulation of gene expressions. These methods build models that can explain a gene's expression level from the regulatory elements (features) on its promoter sequence. Different types of features, such as experimentally verified binding motifs, motifs discovered by computer programs, or transcription factor binding data measured with Chromatin Immunoprecipitation (ChIP) assays, have been used towards this goal. Each type of features has been shown successful in modeling gene transcriptional regulation under certain conditions. However, no comparison has been made to evaluate the relative merit of these features. Furthermore, most publicly available classification tools were not designed specifically for modeling transcriptional regulation, and do not allow the user to combine different types of features.

**Results:**

In this study, we use a specific classification method, decision trees, to model transcriptional regulation in yeast with features based on predefined motifs, automatically identified motifs, ChlP-chip data, or their combinations. We compare the accuracies and stability of these models, and analyze their capabilities in identifying functionally related genes. Furthermore, we design and implement a user-friendly web server called CAGER (Classification Analysis of Gene Expression Regulation) that integrates several software components for automated analysis of transcriptional regulation using decision trees. Finally, we use CAGER to study the transcriptional regulation of *Arabidopsis *genes in response to abscisic acid, and report some interesting new results.

**Conclusion:**

Models built with ChlP-chip data suffer from low accuracies when the condition under which gene expressions are measured is significantly different from the condition under which the ChIP experiment is conducted. Models built with automatically identified motifs can sometimes discover new features, but their modeling accuracies may have been over-estimated in previous studies. Furthermore, models built with automatically identified motifs are not stable with respect to noises. A combination of ChlP-chip data and predefined motifs can substantially improve modeling accuracies, and is effective in identifying true regulons. The CAGER web server, which is freely available at , allows the user to select combinations of different feature types for building decision trees, and interact with the models graphically. We believe that it will be a useful tool to facilitate the discovery of gene transcriptional regulatory networks.

## Background

A major challenge in computational biology is to reveal the *cis*-regulatory logics of gene expression through analysis of high-throughput genomic data, for example, genomic sequences and microarray gene expression data. A common practice is to first identify putatively co-regulated genes by clustering gene expression patterns [1-3], and then search for common motifs from the promoter sequences of these genes [4-6]. However, motif finding methods are often sensitive to noises and usually do not consider combinatorial nature of *cis*-regulation. Furthermore, these methods by themselves do not reveal the actual transcription factors (TFs) that bind to particular sequence motifs.

Recently, many researchers attempted to build quantitative or qualitative models to associate a gene's expression level with regulatory motifs on its promoter sequence. Pilpel et al. [7] explicitly analyzed the combinatorial effects of motif pairs on gene expression profiles and identified many significant motif combinations. Bussemaker et al. [8] and others [9,10] modeled the expression levels of genes as a linear regression of putative binding motifs, and applied feature selection techniques to find the most significant motifs. Hu et al. [11] used decision trees to find motif combinations that best separate two sets of genes. Phuong et al. [12] applied multivariate regression trees to model the transcriptional regulation of gene expressions over several time points simultaneously. Middendorf et al. [13] used an ensemble of decision trees to model gene expression levels by combining putative binding motifs and the expression levels of putative TFs. Simonis et al. [14] combined a string-based motif finding method and linear discriminant analysis to identify motif combinations that can separate true regulons from false ones. Segal et al. [15] and Beer and Tavazoie [16] built probabilistic graphical models, e.g., Bayesian networks, to explain gene expression patterns from motifs. In these models, the predictors (features) are the matching scores of promoter sequences to putative binding motifs, and the predictions (responses) can be continuous or discrete gene expression levels or categorical cluster labels.

The features used in these models generally come from one of the following sources. First, one can use computer programs to automatically find motifs from the promoters of the genes to be modeled [10,11,14-16]. Second, predefined motifs can be obtained independently from sources such as databases of experimentally verified or putative motifs [12,13]. Third, one can enumerate all words up to a certain length as features [8,9]. In addition, TF binding data derived from Chromatin Immunoprecipitation (ChIP) assays [17] have been used as a substitution of motif scores. For example, Banerjee and Zhang [18] directly applied the method of Pilpel et al. [7] to ChIP-chip data to identify TF combinations; Gao et al. [19] replaced the variables in the linear model of Bussemaker et al. [8] with ChIP-chip data and identified significant regulators for many experimental conditions. We recently applied a decision tree method to *S. cerevisiae *ChIP-chip data and identified all known TFs and many interesting TF combinations for yeast cell cycle [20].

Each type of the features discussed above (motifs or binding data) has its advantages and disadvantages in modeling gene transcriptional regulations. As to our knowledge, no comparison has been made to evaluate their relative merits. Modeling accuracy is largely affected by the type of features considered in model construction. If all relevant features are included correctly, many modeling algorithms may have equally high accuracies. On the other hand, if most significant features are omitted, no model can achieve satisfactory accuracy. Furthermore, the inclusion of many irrelevant features may significantly decrease modeling accuracy. Therefore, a comparative study can identify limitations of these feature types, and provide some guidelines and justifications.

Although many classification tools are publicly available (for example, WEKA [21]), most of them are not designed specifically for modeling transcriptional regulation, and are not convenient for biological applications. The only related software is a web server called REDUCE [22], which combines linear regression and feature selection methods to identify significant motifs for specific biological events. It uses enumerated words up to a certain length as features, but does not allow other types of features such as position specific weight matrices [23] or ChIP-chip data to be used. Moreover, the linear model used in REDUCE assumes that each motif contributes linearly to gene expressions, and therefore is unable to represent complex *cis*-regulatory logics such as AND and OR relations [16,24].

In this study, we apply a well-studied classification method, decision tree [25-27], to model significantly up-or down-regulated genes in each of 250 microarray experiments of *S. cerevisiae*. The utilization of decision trees in modeling transcriptional regulation has been explored previously by others [11-13] and in our own research [20]. Here we focus on analyzing the extent to which the expression of these genes can be predicted using different features. We compare the cross-validation accuracies of the models built with different features or feature combinations. We also compare the robustness of these models by introducing noises into training data. Furthermore, we analyze the enrichment of functional categories of the genes that can pass model tests comparing to those fail the tests, and show that decision tree models can be used to detect true regulons. Finally, we present the design and implementation of a user-friendly web interface that combines multiple information sources for automated analysis of gene transcriptional regulations using decision trees. As an example, we also present a case study on the transcriptional regulation of genes in *Arabidopsis thaliana *in response to abscisic acid (ABA) treatment.

## Results and discussion

### Modeling gene transcriptional regulation with decision trees

Here we briefly introduce the modeling of gene transcriptional regulation with decision trees. For a detailed treatment of decision trees, the reader is referred to related literature [20,25-27]. Suppose that there are *N *genes (instances), each of which is represented by a feature vector *F *= *f*_1_, *f*_2_,..., *f*_*m*_ and has a class label *c*, where *f*_*i *_is a real number and *c *is a category. A decision tree is built as follows. Initially, the tree has only one node, the root, which contains all the genes. Then for each node that has no child node,

1. Examine every possible binary split of the genes in the node based on each feature *i*, such that all genes in one subset have *f*_*i *_<*x *and those in the other subset have *f*_*i *_≥ *x*.

2. Select the best split, and create two child nodes that contain the two subsets of genes respectively.

Steps 1 and 2 are then recursively applied to each of the child nodes until no split is possible, or until all genes in the current node have the same label. Finally, some branches of the tree may be pruned to prevent over-fitting. Nodes with or without child node are called internal nodes or leaf nodes, respectively. For examples and biological interpretations of decision trees, see Figure 4 and Figure 5.

Each entry *f*_*i *_in the feature vector of a gene corresponds to the matching score of the gene's promoter to the *i*th binding motif, or the binding data of the *i*th TF to the gene's promoter, depending on the type of features used. A split is equivalent to a test for a gene in the form of, for example, "is the matching score of the gene's promoter to motif A greater than *x*?" or "is the binding affinity of the gene's promoter to TF B greater than *y*?" The exact split point *x *or *y *is determined by maximizing an objective function that reflects the purity of the child nodes. Information gains and gain ratios are two frequently used objective functions [26]. Here we used information gains (see Methods).

The class label of a gene represents a property of the gene that we want to model. For example, one can cluster genes according to expression patterns, and then assign the same label to genes in the same group. Labels can also be derived from other sources such as functional annotations. In this work, we assign labels to genes according to the change of their expressions under certain conditions relative to some reference state (see Experimental setup), and focus our attention on the comparison of different features. This modeling approach is based on the assumption that co-regulated genes very often share common regulatory elements on their promoters. This approach, on the other hand, will not capture post-transcriptional modifications, and will ignore genes that share no regulatory elements with other genes. Note that the underlying assumption may not always be met, since not all co-expressed genes are co-regulated. Moreover, genes may be mislabeled due to noises in their expression data. The purpose is, therefore, to identify rules of thumb for the regulation of the majority genes, while tolerating to some extent the failure in modeling particular genes. As we will see later, decision tree models can indeed be used to detect the true regulons from putatively co-regulated genes.

### Experimental setup

We collected data for 250 microarray experiments on yeast *S. cerevisiae*, of which 77 were for cell cycle [28,29] and 173 were for responses to various stress conditions [30]. We built decision trees for each condition with two classes of instances: positive genes that are differentially expressed (up- or down-regulated) with respect to the reference state, negative genes that are neither up- nor down-regulated. For each experiment, we selected up to 50 up- or down-regulated genes as positive instances and sampled negative instances from non-differentially expressed genes. (See next subsection for selecting candidate genes). Once the genes were selected, we modeled the regulation of up- and down-regulated genes separately. There may be common regulatory motifs shared by up- and down-regulated genes, which may not be revealed if they were modeled together.

**Figure 1:**
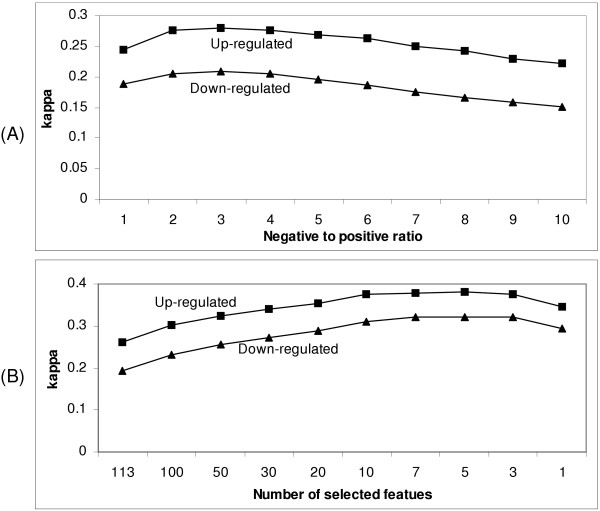
**Effects of class distribution and feature selection on the accuracies of models**. Models were built with ChIP-chip data for up- and down-regulated genes in yeast cell cycle. Each data point is an average of the kappa values of 10 × 77 = 770 models. (A). Effects of class distribution. X-axis ratio of the number of negative instances to the number of positive instances; y-axis: kappa in ten-fold cross-validations; (B). Effects of feature selection. X-axis: number of features selected; y-axis: kappa in ten-fold cross-validations.

Since most genes are not differentially expressed, the number of negative instances is far greater than the number of positive instances. Previous researches have shown that class distribution is an important factor for successful modeling [31]. In general, a skewed class distribution will lead to a degraded modeling accuracy. Therefore, for a set of *n *positive instances, we randomly sampled *μn *instances from all possible negative instances to obtain a desired class distribution. The sampling was repeated 10 times, and each set of sampled negative instances was combined with the set of positive instances to build a decision tree. The accuracy of each model was estimated with a ten-fold cross-validation and measured by the kappa statistic (see Methods). As shown in Figure 1A, the highest accuracy was achieved when *μ *= 3, which is consistent with the results of Simonis et al. [14]. Therefore, we used *μ *= 3 in all subsequent analysis.

We considered three types of features. The first type contained 356 known and putative motifs compiled by Pilpel et al. [7] (referred to as predefined motifs). Of the 356 motifs, 25 were obtained from literature, and the rest were discovered from the promoters of genes sharing similar MIPS functional categories [32]. The second type of features included motifs identified from the promoter sequences of positive genes by the AlignACE program [5] (referred to as auto motifs). The third type of features was derived from the in vivo binding data of 113 transcription factors [17] (referred to as ChIP data). For the first two types of features, each motif was represented as a position specific weight matrix. A promoter was scanned with ScanACE [5] for all motifs in the feature set, and the highest matching score for each motif was used. For the third type of features, the binding affinity of each TF (*p*-value < 0.001, according to [17]) to a promoter sequence was used.

In general, the inclusion of a large number of irrelevant features in training data decreases the accuracy of most classification algorithms. Therefore, feature selection methods are usually applied to reduce the number of features. Most feature selection methods can be categorized as wrappers [33] or filters [34]. A wrapper method searches for a subset of features that maximize the cross-validation accuracy of a given classification algorithm. This strategy is guaranteed to improve the classification accuracy if the same algorithm is used in feature selection and model training. However, it may over-fit to the specific classification algorithm. Furthermore, the method is computationally expensive since many iterations of the classification algorithm need to be executed. In a filter method, features are selected independently of any classification algorithm. Individual features or feature subsets are ranked according to certain scoring functions, and the top ones are selected. This approach is efficient in removing a large number of irrelevant features, but may sometimes eliminate low-ranked, nevertheless important, features. In this study, we used a filter method because of its efficiency in computation. The method ranks individual features according to their information gains and selects the top *d *features (see Methods and [35]). As shown in Figure 1B, the best kappa was achieved with as few as 5 features. This agrees with the fact that a transcriptional regulation only involves a few transcription factors in general. Careful inspections on individual decision trees show that with 5 features, the performance of some trees may be worse than those with more features, due to the loss of some significant features. With 10 features, the modeling accuracies were almost never worse than those with more features. Therefore we used *d *= 10 in all subsequent analysis. The accuracy of each classification model was estimated using a ten-fold cross-validation procedure. First, a training set was randomly divided into ten equal-sized subsets. Each subset was then used in turn as a validation set to test the accuracy of the model built with the other nine subsets. We calculated kappa [36] to measure model accuracies (see Methods). The kappa statistic, written *κ*, measures the agreement between the class labels and the predictions made by the classifier, corrected by the amount of agreement that may be achieved by chance. Therefore, it reflects the extent to which the differential expression between positive and negative genes can truly be explained by the classification model. For example, given a data set containing 20 positive and 80 negative genes, a model that simply guesses all genes as negative agrees with the true labels on 80% of cases, as does another model that makes five mistakes in positive and 15 in negative genes. Taking into account the amount of agreement that we would expect by chance, the value of *κ *is 0.0 for the former model, while 0.47 for the latter.

It is known that *κ *depends on the class distribution and the number of categories in the test data [37]. This, however, was not a problem in our case, since the models in our test all had binary classes and the same class distributions. Another difficulty associated with *κ *is its lack of interpretability, although some relations between *κ *and model quality have been suggested [36]. Therefore, in addition to *κ*, we calculated sensitivity (*SS*) and specificity (*SP*) for each model (see Methods). *SS *is the proportion of positive genes that are correctly predicted by the model, i.e., the proportion of up- or down-regulated genes that can be explained by the regulatory elements identified. *SP *is the proportion of negative genes that are correctly predicted by the model, and 1 - *SP *represents the proportion of negative genes that cannot be separated from the positive genes based on the regulatory elements.

### Methods for identifying DEG candidates

Microarray data are noisy and often measured with limited or no replication, which makes the identification of differentially expressed genes (DEGs) difficult. In most early microarray analysis, a fixed fold-change threshold (generally two-fold) was used to identify DEGs, while more sophisticated methods have emerged recently [38-41]. Although it is not the focus of this paper, we considered and compared several different DEG identification methods to show that our conclusions on the classification models are unlikely to be affected by the specific DEG identification method used.

For the first method (referred to as the vanilla method), we downloaded the transformed and normalized log ratios for the 250 microarray experiments. The data set had been corrected for background noises, and globally normalized by constant factors such that the mean log_2_(*cy*5/*cy*3) value is zero within each slide [40]. For expression data in cell cycle conditions, the log ratios were further normalized such that the mean log ratio for each gene across all cell cycle conditions is zero [28]. Several rules were also applied to remove spurious data points [28]. For each column (condition) of this data set, we selected the genes with log_2_(*cy*5/*cy*3) ≥ 1 (more than two-fold induction) as up-regulated genes. In cases there were more than 50 up-regulated genes, we selected the top 50 with the highest fold changes, or until ties were broken. Likewise, down-regulated genes with log_2_(*cy*5/*cy*3) ≤ - 1 were selected. Genes with |log_2_(*cy*5/*cy*3)| ≤ 0.6 (less than 1.5 fold expression change) were considered as non-DEGs and were used to sample negative instances. Note that we intentionally used two different thresholds for DEGs and non-DEGs, in order to exclude genes whose labels may be ambiguous.

For the second to fifth methods, we downloaded the raw intensity data. However, we only found raw data for 216 of the 250 conditions, and it was sometimes unclear how to match the name of a raw data file with a column in the log ratio data. The intensity data were background corrected, without any other normalization or transformation. We removed low quality data points that were annotated by the authors with failed status or non-zero flags.

The second approach (referred to as the global normalization method [40]) was similar to the vanilla approach, except that no per-gene normalization was made. The third approach (referred to the lowess normalization method) was similar to the second approach, except that within-slide normalization factors were intensity dependent, obtained through a locally weighted regression approach [40]. The fourth method (referred to as the vsn method) transformed the intensities with a generalized logarithm function, in order to stabilize the variances which were originally intensity dependent [39]. We applied the same thresholds as in the vanilla approach to the transformed data. The fifth method (referred to as the EDGE method) quantified the heteroscedasticity as a function of intensity and then taking it into account to identify DEGs [41]. The EDGE method assigned each gene a *p*-value based on its distance from the line of equivalence (*cy*5 = *cy*3), corrected for multiple tests. We ranked genes according to their corrected *p*-values, and selected up to 50 up-regulated or down-regulated genes with false discovery rate (FDR) < 0.002 [42]. The FDR threshold was used to ensure that the number of DAGs selected by EDGE was approximately equal to those chosen by the other approaches. It had no impact for most experiments, where all top 50 genes had FDRs less than 0.002. Genes with FDR > 0.5 were considered as non-DEGs. The programs for vsn and EDGE were obtained from their original authors.

We used each of the five methods to select DEGs and non-DEGs for each microarray. DEG sets with less than 20 genes were not used. Table 1 lists the average group size and the number of DEG sets identified by each method. The accession numbers for the genes in each set can be viewed on the supplementary website [43].

**Figure 2:**
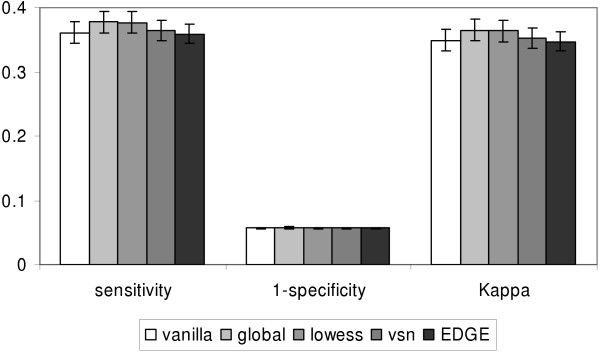
**The accuracies of models built for DEGs identified by different methods**. The three groups of bars show the mean *SS*, 1 - *SP *and kappa values of the models built for genes selected by five different DEG identification methods. The error bars represent individual 95% confider interval for the means.

For each set of positive genes (up- or down-regulated), we randomly sampled threefold negative genes from the corresponding non-DEGs to construct a decision tree and performed a ten-fold cross validation. The random sampling was repeated for ten times for each DEG set. The cross-validation accuracies for all models are included in Supplementary Table 1 (see Additional file 1). Figure 2 shows the values of *κ*, *SS *and 1 - *SP *averaged across all gene sets obtained by each method. The five different methods showed similar accuracies in all three measures. Although two of the methods (global and lowess) seemed to have slightly better *SS *and kappa, the difference is not significant. We therefore restricted our subsequent analysis on the DEGs identified using the vanilla method, which resulted in the largest number of DEG groups.

### A comparison of the prediction power of different features

We compared the cross-validation accuracies of the models built with three types of features that we discussed early: ChIP-chip data, predefined motifs and auto motifs. The combination of ChIP-chip data with predefined motifs was also tested. For each type of features or feature combinations, 446 × 10 = 4460 decision tree models were built (446 sets of DEGs and 10 sets of negative genes randomly sampled for each set of DEGs). We randomly exchanged the labels for positive and negative genes to serve as controls. Note that we carried out two types of cross-validations for models built with auto motifs. In the first method, promoter sequences of genes in both training data and test data were combined to find motifs. In other words, motifs were identified from all positive genes, and the same set of motifs was used to train models for each fold in a cross-validation. In the second method, genes were first divided into ten subsets without constructing the actual feature vectors. A subset was chosen for testing, and the other nine subsets were used for motif finding. In other words, motifs were identified from only the training genes, and a different set of motifs was used to train models for each fold in a cross-validation. The second method provided a more stringent estimation of the generalization accuracy of a model, since it completely hided the test data from the learning algorithm until they were tested. The first method, however, was used in several previous studies [11,16], probably because it is simple to implement and convenient to test. Here we analyzed the results of both cross-validation methods to compare auto motifs with other feature types. In the next two subsections, we used only the first method to show other aspects of the models based on auto motifs.

**Figure 3:**
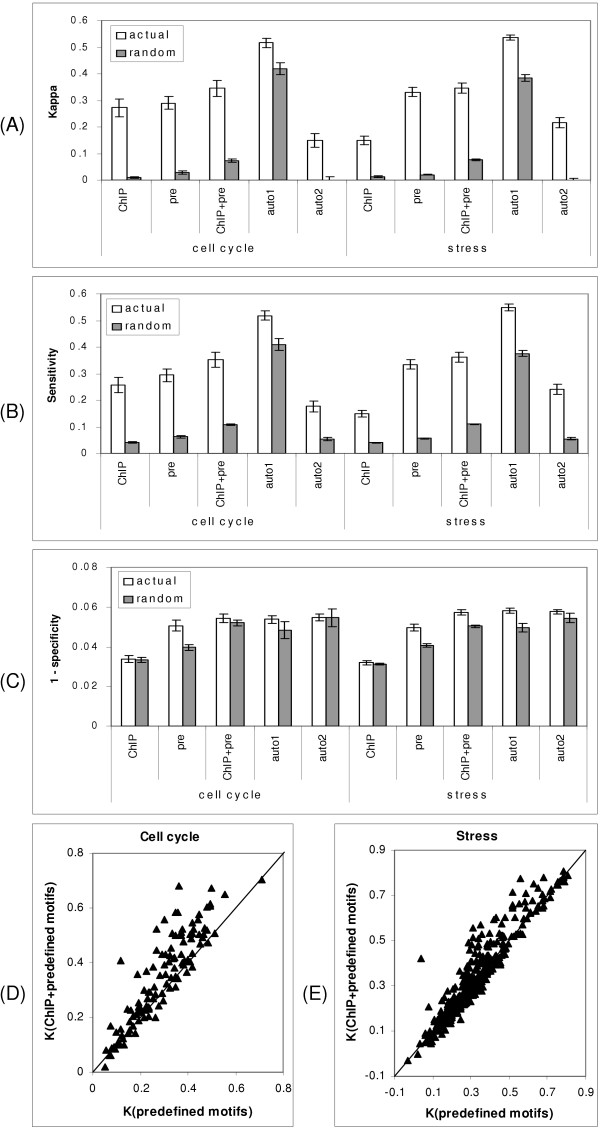
**The accuracies of models built with different features**. (A), (B) and (C) show kappa, *SS *and 1 - *SP *for models built with different features, respectively. ChIP: ChIP-chip data, pre: predefined motifs, auto: automatically identified motifs using AlignACE. The first cross-validation method was used for models in autol and the second cross-validation method was used for models in auto2 (see text). White bar: average accuracy of models for true DEGs. Grey bar: average accuracy of models for random genes. The error bars represent individual 95% confident interval for the means. (D) and (E) show kappa values for models built with different types of features for stress conditions and cell cycle conditions, respectively. X-axis: kappa for models built with predefined motifs; y-axis: kappa for models built with a combination of ChIP-chip data and predefined motifs.

A complete list of the cross-validation accuracies of models for each microarray experiment is included in Supplementary Table 2 (see Additional file 2). The mean cross-validation accuracies of models for genes in cell cycle and stress conditions are shown in Figure 3.

As shown in Figure 3A, when the first cross-validation method is used, the models using auto motifs have the highest kappa values (~0.53) among the three individual feature types. However, it is important to note that these models also have the highest kappa values on randomly selected genes (~0.4).

Furthermore, the accuracies measured by the second cross-validation method are much lower: the average kappa values are 0.15 for models in cell cycle and 0.22 for models in stress response experiments, and are approximately zero for models of randomly selected genes. Therefore, the first method considerably over-estimates the accuracies of the models built with auto motifs. This is because that, with the first cross-validation method, the feature set contains some information about the test instances, even though the models are built only on training instances. Consequently, although the results reported in previous studies utilizing automatically identified motifs [11,16] may still be valid qualitatively, the exact accuracies may need to be re-evaluated. Nevertheless, an apparent advantage of using automatically identified motifs is that it may be able to discover new features not included in predefined motifs and ChIP data.

In cell cycle experiments, the models using ChIP data or predefined motifs have similar kappa values (0.27 and 0.29, respectively; *p*-value = 0.15 in a paired *t*-test). In contrast, in stress response experiments, the models using ChIP data alone have very low kappa values than that using predefined motifs (0.15 vs. 0.33, *p*-value = 10^-47 ^in a paired *t*-test). The ChIP data used in this study were measured under normal cell growth conditions. However, it is known that TF binding may change with environmental conditions [44]. While the cell cycle expression data were measured under conditions relatively similar to normal cell growth conditions, stress treatment dramatically changes the environmental conditions and thereby alters the binding of TFs. It is thus expected to observe lower prediction accuracies in stress response experiments than in cell cycle experiments when using ChIP data alone.

Figure 3B and Figure 3C show the mean *SS *and 1 - *SP *for the models built with different features.

Figure 3B for *SS *shows almost identical trends as Figure 3A, which means that with combined features, the models can better explain the co-regulation of the genes. On the other hand, Figure 3C shows that models built with ChIP data have higher specificity than those built with predefined or auto motifs (*p*-value = 10^-68 ^in a paired *t*-test between ChIP data and predefined motifs). This can be explained by the fact that ChIP data are more reliable than motif scores as an indicator of co-regulations, since ChIP data explicitly measure the binding affinities of gene promoters to TFs. There are also other advantages in using ChIP-chip data as features. For example, the number of features is well bounded by the number of TFs, which is estimated to be around 200 in yeast [44], comparing to thousands of putative binding motifs. As a result, the correlations and redundancies among features are low in ChIP-chip data, which makes it possible to build simple models for better interpretability. Furthermore, the models built with ChIP-chip data directly suggest regulatory relations between TFs and genes, which can be used to construct regulatory networks. Our results indicate that, however, the conditions of microarray experiments and ChIP assays must be considered with special care.

In cell cycle experiments, the models built with a combination of predefined motifs and ChIP data have substantially better kappa values than those with ChIP data or predefined motifs alone (Figure 3D). The *p*-value is 10^-14 ^in a paired *t*-test for results of ChIP data (0.27) and combined features (0.34), and 10^-12 ^for predefined motifs (0.29) and combined features (0.34). In stress experiments, the kappa values for models based on combined features (0.35) are only marginally better than that for predefined motifs (0.33), due to limited usage of ChIP data as pointed out above. Nevertheless, the models using combined features perform significantly better (improvement of kappa > 0.1) in 33 cases, while the models using predefined motifs are never better by more than 0.1 in kappa (Figure 3E). A paired *t*-test yields a *p*-value 10^-5^, which is still statistically significant. Putative binding motifs and ChIP data represent two distinct and complementary sources of evidence of regulation. Therefore, a combination of them can provide a better discriminative power than either of them does.

Since the role of decision tree models is exploratory, there is no need to be restricted to any specific type of features. Indeed, one should try a variety of them to identify the most relevant features for a particular set of genes. A good strategy in choosing feature types is probably as follows: first use a combination of TF binding data and predefined motifs for modeling; if a model with good cross-validation accuracy can not be found, then consider using automatically identified motifs. Obviously when predefined motifs or TF binding data are unavailable or insufficient, using automatically identified motifs as features is the only option.

### Stability of models

To analyze the stability of decision tree models, we introduced some noises into positive training data and tested whether the models can separate the true positives from the noises. For each microarray experiment we randomly selected 5 to 50 negative genes and deliberately mislabeled them as positive. The original data and the additional fake positive instances were then combined to build a decision tree model. We compared the results of the new models to the results of the original models in ten-fold cross-validations and counted the numbers of losses, rescues and false positives (FPs). According to [14], a *loss *is a positive instance that is mis-classified in the noisy data but correctly classified in the original data. A *rescue *is a positive instance that is correctly classified in the noisy data but mis-classified originally. An *FP *corresponds to a newly added noise gene that is classified as a (false) positive gene.

As shown in Table 2, the models built with predefined motifs are more robust than the models built with auto motifs. Even at 100% noise level (50 randomly selected genes added), almost 90% of the introduced noises  can be correctly filtered out by the models built with predefined motifs. In comparison, the models built with auto motifs can only filter out ≈ 75% of the noises. This is because the motif finding program was distracted by the wrongly labeled genes so that the discovered motifs became less effective to discriminate the true positives from false ones.

Furthermore, the predictions made by the models built with predefined motifs are more stable, as reflected by the smaller values of *loss *+ *rescue *in Table 2. The addition of a small amount of noises may result in, dramatic change to the predictions by the models built with auto motifs. When as few as 5 noisy instance were added, the models based on auto motif changed their predictions by  ≈ 58%, while the models based on predefined motif changed their predictions by only  ≈ 30%.

### Functional enrichment in correctly classified positive genes

As mentioned early, there may be errors in the labels of training instances, i.e., some genes assigned with the same label may actually not be co-regulated. Here we test decision tree models' capabilities of identifying "true" regulons from such genes. Given a set of putatively co-regulated genes as positive instances and randomly sampled genes as negative instances, we used a leave-one-out strategy to predict which of the positive genes are indeed co-regulated. To this end, a positive instance was removed from the training data, and tested on the decision tree model built without it. This was repeated for each positive gene. Consequently, the positive genes were categorized into two groups: those that were predicted as true positives and those as negatives, which we refer to as Gp and Gn, respectively. We hypothesized that the genes in Gp are more functionally coherent than the genes in Gn, since according to the decision tree model, the genes in Gp share some common regulatory elements. To test this hypothesis, we counted the number of Gene Ontology (GO) functional categories [45] that are statistically enriched in each group with a false discovery rate < 0.05 (see Methods). The above procedure was applied to the up- and down-regulated genes in each of the 250 microarray experiments and the average results were calculated. As shown in Table 3, the models built with predefined motifs and with auto motifs both succeed in retaining functionally related genes and filtering out unrelated genes. For example, in the models built with predefined motifs for up-regulated genes in cell cycle, there are in average 15 enriched GO categories in 17 Gp genes, while there are only 8 enriched GO categories in 27 Gn genes. A similar trend can be observed for down-regulated genes and for the models built with auto motifs. A paired *t*-test between 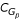 and 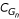 combining all cases yields a *p*-value 10^-43^.

Furthermore, we tested whether a similar degree of functional enrichment can be achieved without decision tree models. Suppose a decision tree model predicted 25 out of 50 positive genes as true positives, and GO analysis showed that these 25 genes were functionally more coherent than the remaining 25 genes. We want to know whether there is another way to select 25 genes that have higher degree of functional enrichment than those predicted by decision trees. For example, we may simply pick genes that are top ranked according to their expressions. We used the following procedure to test this hypothesis. For each set of up-or down-regulated genes, *A*, which has *p *predicted true positive genes, we selected *p *top ranked genes from *A *based on the absolute log ratios of their expressions. We denoted this set of genes as Gp', which has the same number of genes as Gp, and counted the number of enriched GO categories in it. The average results over all microarray experiments are shown in the 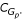 column of Table 3. As shown, the genes in Gp contain more enriched functional categories than genes in Gp'(). A paired *t*-test between 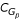 and 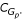 combining all cases has a *p*-value 10^-16^. Therefore, we can conclude that both models built with auto motifs and with predefined motifs are more effective in selecting functionally related genes than the naive differential expression model.

### CAGER web server

Several previous studies have shown that decision tree is a valuable tool in analyzing transcriptional regulation of gene expressions [11-13,20]. Although there are many publicly available software packages for building decision trees (for example, [21,25,26]), they are not specifically designed for biological applications, and are not convenient for biologists to use. Therefore, to make a good use of the results from this study, we designed and implemented a user-friendly web server and interface for building decision trees to analyze transcriptional regulation. The server integrates several software components that allow the user to select from different types of features and to interact with the constructed models.

**Figure 4:**
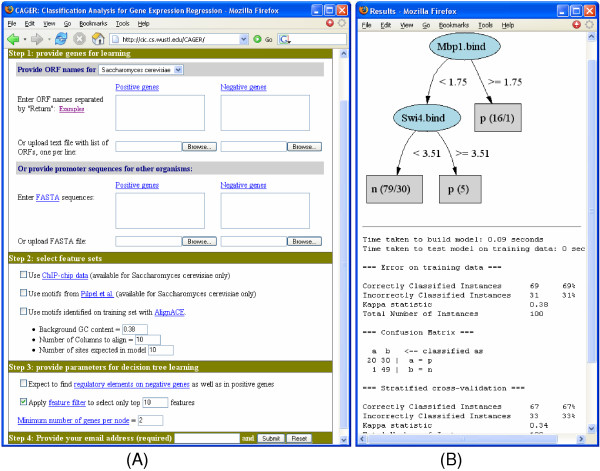
**Screenshots of the input and output interfaces of CAGER**. (A). The input consists of four steps. First, the user provides ORF identifies or promoter sequences for positive and negative genes. Second, the user selects a type of features or some of their combinations. Third, the user can change parameters for decision tree learning. The user then provides an email address for notification of results and finally submits the job. (B). On the top of the output page is a graphical representation of a decision tree. Each oval of the decision tree represents an internal node and each box represents a leaf node. The text inside an internal node is the name of a feature, and the text associated with an edge is a test of the feature. The text inside a leaf node gives the predicted label (p: positive, n: negative) for genes inside the leaf, and the number of supporting and counter-instances, if any. A path from the root to a leaf node defines a possible regulatory rule. For example, the rightmost path can be read as "if the binding affinity of Mbpl to a gene's promoter is at least 1.75, the gene is positive (i.e., up-regulated under the condition that the decision tree models)." The numbers "16/1" enclosed in parenthesis means that 16 training instances have feature *Mbpl_bind *≥ 1.75, of which 15 are positive and one is negative. On the bottom of the output page are related statistics for training and cross-validation of the decision tree model.

The interface for user inputs is shown in Figure 4A. To submit a job to CAGER, the user is first asked to provide positive and negative genes as either ORF identifies for one of the supported organisms, which currently includes yeast *S. cerevisiae *and *Arabidopsis thaliana*, or promoter sequences in FASTA format. These can be copy-pasted to the web form or uploaded from files in the user's local computer. Given ORF identifiers, the promoter sequences in a supported genome are retrieved automatically from a local database. Second, the user specifies some feature sets, which may be ChIP-chip data for yeast *S. cerevisiae *[17], predefined motifs from Pilpel et al. [7], motifs automatically identified from the promoter sequences by AlignACE [5], or a combination of them. The user can also specify whether features should be identified from negative instances as well as from positive instances, whether feature filters should be used, and the minimum number of instances per node. Finally, the user fills in his or her email address and submits the job. After a job is completed, the user will be notified by email for instructions about how to access the results.

On the output page, a decision tree is displayed as a portable network graph (PNG), along with related statistics for the tree in training and cross-validation processes (Figure 4B). The text inside an internal node of the tree gives the name of a feature, and the text inside a leaf node shows the predicted label for genes inside the node, as well as the number of supporting and counter-instances for the prediction. Each node of the decision tree can be clicked to show some details. For example, if an internal node contains a feature derived from ChIP-chip data for a TF in yeast, clicking on it leads the user to SGD [46] for detailed information about the TF. If the feature is a binding motif, a click opens a new window to display the sequence logo [47] and the position specific weight matrix of the motif. A click on a leaf node brings up a window for displaying the identifiers of the positive and negative genes in the leaf.

### Application to ABA-responsive genes in *Arabidopsis*

Here we show an example of using the web server to study the regulation of genes expressed in response to abscisic acid (ABA) in *Arabidopsis*. ABA is a phytohormone that plays important roles in many stages of plants, such as seed development and stress responses (see [48] for a review). Seki et al. [49] identified about 250 genes in *Arabidopsis *that are induced by at least 5-fold after ABA treatment. Since *Arabidopsis *is one of the supported organisms in our current server, its promoter sequences are available in our database.

**Figure 5:**
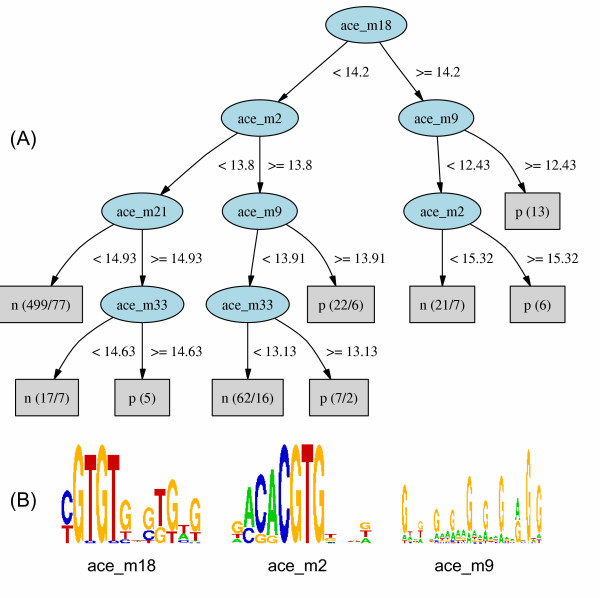
**Decision tree and motifs learned for ABA-responsive genes in *Arabidopsis***. (A). A decision tree model learned for the transcriptional regulation of abscisic acid-induced genes in *Arabidopsis*. (B). Sequence logos of the three most significant motifs used by the decision tree model.

Therefore, we provided as positive instances the list of ORF names corresponding to the up-regulated genes, and as negative instances a list of randomly selected genes that are not up-regulated. Promoter sequences were retrieved for 152 of the positive genes. We used auto motifs identified from these promoters as features. The decision tree and the sequence logos for the most interesting motifs are shown in Figure 5. AlignACE identified a total of 37 motifs with default parameters, five of which were selected by the decision tree (Figure 5A). Three motifs, ace_m2, ace_m9, and ace_18 (Figure 5B) together correctly classified 35 (= 13 + 6 + 22 - 6) positive genes (the rightmost three leaves labeled with 'p'), while as many as 107 positive genes were classified as negative. This may be due to the fact that ABA triggers a lot of down-stream responsive genes, many of which are not co-regulated with direct targets of ABA. The motif ace_m2 has a conserved CACGTG core, which is very close to the known ABA responsive elements (ABREs) identified in many other plants [50-56]. It is known that an ABRE often functions together with a coupling element (CE), but the consensus sequence of CE in *Arabidopsis *is elusive. Here, the decision tree suggests that ace_m18 and ace_m9 may be two possible CEs. Motif ace_m18 has a CGTGTG core which partially resembles the CE in rice *OSEM *gene (GACGCGTGTC) [57], and CE3 in barley *HVA1 *gene (ACGCGTGTCCTC) [55]. Note that ace_m18 has a weak second copy of the GTGTG core, whicl may be important for enhanced binding activity. Motif ace_m18 is also remotely similar to CE3 in maize *RAB28 *gene (ACGCGCCTCCTC) [53], although in maize the CGTGTG core is replaced by CGCGCC. Motif ace_m9 is a weak but significant motif that consists of a series of G's separated by one or two A's. This motif is not similar to any known motif in the database of Plant *Cis*-acting Regulatory DNA Elements (PLACE) [58], and therefore may be a new motif for *Arabidopsis*.

## Conclusion

In this research, we compared the effect of using different features to study transcriptional regulation of gene expressions by classification methods. We considered features based on ChIP-chip data, predefined motifs, automatically identified motifs, and their combinations. We found that TF binding data from ChIP assays are effective in modeling gene expressions only under the same conditions where ChIP-chip experiments were conducted. Our results also indicate that many previous studies may have over-estimal the cross-validation accuracies of models built with automatically identified motifs. Furthermore, the models built with automatically identified motifs are not robust with respect to noises, comparing to those built with predefined motifs. A combination of ChIP-chip data with predefined motifs seems to be superior to either one of them applied separately.

We also showed that the positive genes correctly predicted by decision tree models are more functionally related than those that are not correctly predicted. Therefore, decision tree models can be used to refine putative regulons and detect new genes of a regulon. Simonis et al. [14] have showed this by testing on known regulons, while we confirmed this through analyzing the functional enrichment of predicted regulons. We presented a web service that integrates motif finding and decision tree learning for analyzing transcriptional regulation of gene expressions. Its usefulness was illustrated with an example of studying the regulation of ABA-responsive genes in *Arabidopsis*. We identified two motifs that are similar to known ABA-responsive elements (ABREs) and coupling elements (CEs), and suggested a new CE, which may deserve further studies. As demonstrated by the example, the web interface combines a number of software components and hides most specific parameters from the user, while still allows some flexibilities. The graphical representation of a decision tree makes it easy to visualize and extract significant regulatory rules. We believe that it can significantly reduce the learning curve for those who are interested in applying classification methods to analyzing transcriptional regulation, and will be a useful tool to facilitate the discovery of transcriptional regulatory networks by combining multiple information sources.

## Methods

### Datasets

Yeast gene expression data were downloaded from from Expression Connection [59]. ABA-induced gene expression data for *Arabidopsis *were obtained from [49]. ChIP-chip data for yeast transcription factors were downloaded from [60]. Putative binding motifs for yeast genome were obtained from [61]. Upstream sequences of yeast and *Arabidopsis *ORFs were obtained from RSA-tools [62] by retrieving up to 800 and 1500 bases, respectively, from translation start sites. Overlaps with other ORFs were truncated, and upstream sequences shorter than 100 bases were discarded. For yeast ORFs, the upstream sequences were used as promoters. For *Arabidopsis *ORFs, the upstream sequences were used as inputs to a promoter prediction program, TSSP [63], to predict transcription start sites (TSSs). Promoters of *Arabidopsis *ORFs were defined as from 350 bases upstream to 50 bases downstream relative to the predicted TSSs, where most known ABREs and CEs were discovered.

### Feature filters and decision tree learning

Given a training set, features were ranked according to the restricted information gains that can be achieved by using individual features to separate positive and negative instances. Suppose that there are *p *positive instances and *n *negative instances, and by selecting a splitting point *x *on feature *f*_*i*_, the numbers of positive and negative instances on the left (*f*_*i *_<*x*) and right (*f*_*i *_≥ *x*) child nodes are *p*_1_, *n*_1_, *p*_2_, and *n*_2_, respectively. The restricted information gain  due to feature *f*_*i *_with respect to this split can be calculated by:



where *I*_*gain *_is the normal information gain computed from entropies [26]. The restriction to the information gain calculation ensures that a selected feature is more over-represented in positive instances than in negative instances, which is necessary since negative instances were chosen randomly and therefore should not be co-regulated. Features were ranked by  and the top *d *features were selected for decision tree building (*d *is denned by the user and is default to 10 in this study). Decision trees were built with C4.5 algorithm [26], except that  instead of *I*_*gain *_were used.

### Measuring model accuracy

Let *TP*, *TN*, *FP*, and *FN *be the numbers of true positive, true negative, false positive and false negative predictions made by a binary classifier, respectively, and *N *= *TP *+ *TN *+ *FP *+ *FN*. Sensitivity is defined as . Specificity is defined as . The kappa static *κ *of the classifier is defined as , where  is the percentage of correctly predicted instances, and *C *is the expected percentage of instances that a classifier can predict correctly by chance.



Note that when *A *= 100% and *C *≠ 100%, *κ *= 1.0, corresponding to a perfect classifier; when *A *≤ *C*, *κ *≤ 0, meaning that the classifier does not perform better than random guessing.

### GO functional enrichment

GO annotations were retrieved from SGD (version September 2004) [46]. Go functional enrichment were calculated with accumulative hyper-geometric distribution. GO::TermFinder perl module [64] was used to search for significantly enriched functional categories with a false discovery rate (FDR) < 0.05 [42].

### Software and implementation

Motifs were identified with AlignACE [5] and scanned against promoter sequences with ScanACE [5]. Decision trees were built with the J48 program, which is a java implementation of the C4.5 decision tree learning algorithm [26], included in the WEKA machine learning package [21]. Decision trees were drawn with the dot program in Graphviz 1.0 [65] and displayed with webdot in the same package. Sequence logos of motifs were drawn with the seqlogo program [47]. The CAGER web service was implemented in perl and run on an apache web server with dual AMD Athlon 1600 MHz CPUs and 2 GB of RAM.

## Authors' contributions

JR designed the experiments, performed the analysis, and prepared the manuscript. WZ provided essential guidance during the study and revised the manuscript critically. All authors read and approved the final manuscript.

## List of abbreviations

TF: transcription factor. ChIP: Chromatin Immunoprecipitation. GO: gene ontology. ABA: abscisic acid. ABRE: ABA-responsive element. CE: coupling element. ORF: open reading frame. DEG: differentially expressed gene. SS: sensitivity. SP: specificity. FDR: false discovery rate. TP: true positive. FP: false positive. TN: true negative. FN: false negative.

## Supplementary Material

Additional File 1 This Excel file contains Supplementary Table 1 that lists the cross-validation accuracies of decision tree models built for genes selected by different DEG identification methods. Click here for file

Additional File 2 This Excel file contains Supplementary Table 2 that lists the cross-validation accuracies of decision tree models built with different types of features. Click here for file

## Figures and Tables

**Table 1:** Statistics of DEG sets identified by different methods. The ranges represent one standard deviation from the means.

	EDGE	global	lowess	vanilla	vsn
Number of down-regulated sets	215	202	194	223	204
Number of down-regulated sets	215	210	200	223	195
Number of genes per set	48 ± 3	47 ± 8	45 ± 6	49 ± 7	46 ± 6

**Table 2:** Stability of decision tree models. Up: the models built for up-regulated genes. Down: the models built for down-regulated genes. Noise: the number of noisy instances added into the training set. TP: the number of true positive genes predicted by models built on the original data. TP': the number of true positive genes predicted by models built on the noisy data. Loss: the number of positive instances correctly classified in the original data but mis-classified in the noisy data. Rescue: the number of positive instances correctly classified in the noisy data but mis-classified originally. FP: the number of newly added noise genes classified as positive. Each value is an average across 223 up-regulated or 223 down-regulated gene sets. The standard errors for loss, rescue and FP are all less than 0.2.

		Predefined motifs					Auto motifs				
		
	Noise	TP	TP'	Loss	Rescue	FP	TP	TP'	Loss	Rescue	FP
Up	0	16.6	16.6	0.0	0.0	0.0	26.4	26.4	0.0	0.0	0.0
	5	16.6	17.1	2.5	2.4	0.6	26.4	27.7	7.8	7.6	1.5
	10	16.6	17.4	3.6	3.2	1.1	26.4	28.9	8.1	7.7	2.9
	15	16.6	17.7	4.0	3.5	1.6	26.4	30.3	8.2	7.7	4.4
	20	16.6	18.8	4.2	4.1	2.4	26.4	31.7	8.3	7.8	5.8
	25	16.6	19.3	4.5	4.2	3.0	26.4	32.6	8.5	7.8	6.8
	50	16.6	21.2	5.8	4.6	5.7	26.4	37.8	9.1	7.2	13.3

	0	19.1	19.1	0.0	0.0	0.0	27.9	27.9	0.0	0.0	0.0
Down	5	19.1	19.6	2.0	2.0	0.5	27.9	29.1	7.3	7.0	1.5
	10	19.1	20.5	2.7	2.9	1.1	27.9	29.6	8.0	6.8	2.9
	15	19.1	20.7	3.2	3.2	1.6	27.9	30.6	8.1	6.6	4.1
	20	19.1	20.9	3.8	3.5	2.1	27.9	30.9	8.8	6.5	5.3
	25	19.1	21.2	4.1	3.6	2.6	27.9	32.8	8.5	6.6	6.9
	50	19.1	22.6	5.3	3.6	5.2	27.9	37.8	9.7	6.2	13.4

**Table 3:** Functional enrichment in correctly predicted positive genes. Predefined motifs: the models built with predefined motifs. Auto motifs: the models built with auto motifs. Up: the models built for up-regulated genes. Down: the models built for down-regulated genes. *#Gp*: the number of Gp genes (positive genes that are predicted as positive by a model). *#Gn*: the number of Gn genes (positive genes that are predicted as negative by a model). 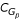: the number of enriched functional categories in the set of Gp genes. 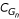: the number of enriched functional categories in the set of Gn genes. 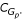: the number of enriched functional categories in the set of Gp' genes (see text). The sample sizes for up- and down-regulated genes in cell cycle are 56 and 59, respectively, and 167 and 164 in stress conditions. The ranges represent individual 95% confidence interval for the means.

Model			*#Gp*	*#Gn*	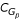	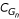	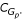
Predefined motifs	Cell cycle	Up	17.2 ± 2.2	27.1 ± 2.7	15.1 ± 2.9	8.2 ± 2.6	12.3 ± 3.5
		Down	14.0 ± 2.1	31.0 ± 2.5	14.5 ± 3.7	9.6 ± 3.4	8.4 ± 3.3
	Stress	Up	16.4 ± 1.0	33.1 ± 1.1	13.4 ± 2.3	10.6 ± 1.8	10.3 ± 1.9
		Down	21.0 ± 2.0	30.7 ± 1.7	17.1 ± 2.4	7.8 ± 1.7	14.7 ± 2.3

Auto motifs	Cell cycle	Up	24.9 ± 1.8	19.3 ± 1.8	16.9 ± 3.2	7.9 ± 2.5	13.9 ± 3.2
		Down	23.6 ± 1.9	21.4 ± 1.8	15.6 ± 3.7	8.4 ± 2.9	11.8 ± 3.5
	Stress	Up	27.0 ± 0.8	22.6 ± 0.8	16.2 ± 2.3	9.1 ± 1.9	15.2 ± 2.4
		Down	29.4 ± 1.3	22.2 ± 1.1	18.2 ± 2.3	6.6 ± 1.5	16.4 ± 2.4
